# *catena*-Poly[1,1′-dimethyl-4,4′-(ethane-1,2-di­yl)dipyridinium [lead(II)-tri-μ-iodido-lead(II)-tri-μ-iodido]]

**DOI:** 10.1107/S160053681102006X

**Published:** 2011-06-04

**Authors:** Guohai Xu, Xiyun He

**Affiliations:** a Key Laboratory of Jiangxi University for Functional Materials Chemistry, Department of Chemistry and Life Science, Gannan Normal University, Ganzhou, Jiangxi 341000, People’s Republic of China

## Abstract

The title compound, {(C_14_H_18_N_2_)[Pb_2_I_6_]}_*n*_, consists of discrete 1,1′-dimethyl-4,4′-(ethane-1,2-di­yl)dipyridinium cations and one-dimensional [Pb_2_I_6_]_*n*_ anions. The organic cation has an inversion center at the mid-point of the ethane C—C bond. In the anion, the Pb^II^ atom is coordinated by six I atoms in a distorted octa­hedral geometry. The I atoms bridge the Pb^II^ atoms into a polymeric chain running along [001]. These inorganic chains are separated by the isolated organic cations.

## Related literature

For general background to the applications of metal halides, see: Jin *et al.* (2011 ▶); Manjunatha *et al.* (2011 ▶). For bond-length data, see: Lemmerer & Billing (2006 ▶).
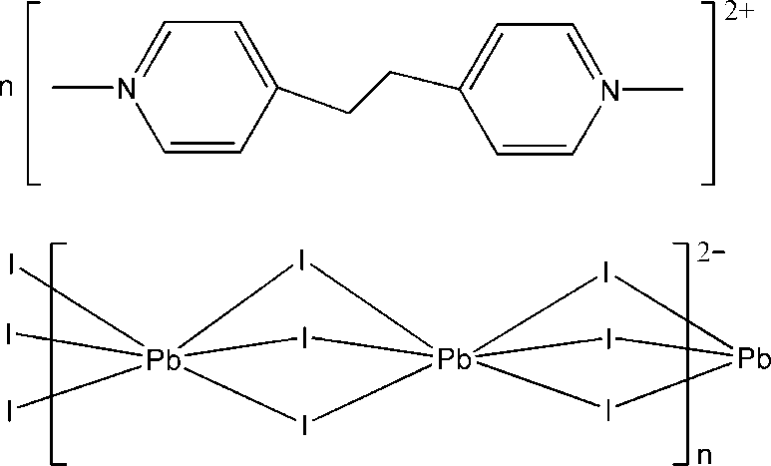


## Experimental

### 

#### Crystal data

(C_14_H_18_N_2_)[Pb_2_I_6_]
*M* *_r_* = 1390.08
Monoclinic, 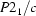

*a* = 10.120 (6) Å
*b* = 17.575 (10) Å
*c* = 8.025 (4) Å
β = 101.239 (10)°
*V* = 1399.9 (13) Å^3^
*Z* = 2
Mo *K*α radiation
μ = 18.63 mm^−1^
*T* = 296 K
0.25 × 0.20 × 0.19 mm

#### Data collection

Bruker APEXII CCD diffractometer
Absorption correction: multi-scan (*SADABS*; Bruker, 2001 ▶) *T*_min_ = 0.016, *T*_max_ = 0.030
22768 measured reflections
3425 independent reflections
2838 reflections with *I* > 2σ(*I*)
*R*_int_ = 0.038

#### Refinement

*R*[*F* ^2^ > 2σ(*F* ^2^)] = 0.022
*wR*(*F* ^2^) = 0.046
*S* = 1.02
3425 reflections
109 parameters
H-atom parameters constrained
Δρ_max_ = 0.79 e Å^−3^
Δρ_min_ = −1.16 e Å^−3^

### 

Data collection: *APEX2* (Bruker, 2007 ▶); cell refinement: *SAINT* (Bruker, 2007 ▶); data reduction: *SAINT*; program(s) used to solve structure: *SHELXTL* (Sheldrick, 2008 ▶); program(s) used to refine structure: *SHELXTL*; molecular graphics: *SHELXTL*; software used to prepare material for publication: *SHELXTL*.

## Supplementary Material

Crystal structure: contains datablock(s) global, I. DOI: 10.1107/S160053681102006X/hy2435sup1.cif

Structure factors: contains datablock(s) I. DOI: 10.1107/S160053681102006X/hy2435Isup2.hkl

Additional supplementary materials:  crystallographic information; 3D view; checkCIF report

## Figures and Tables

**Table 1:** Selected bond lengths (Å)

Pb1—I1	3.2296 (13)
Pb1—I1^i^	3.2214 (13)
Pb1—I2	3.2311 (11)
Pb1—I2^ii^	3.1724 (11)
Pb1—I3	3.2435 (12)
Pb1—I3^ii^	3.2073 (12)

Symmetry codes: (i) 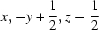; (ii) 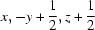.

**Fig. 1.**
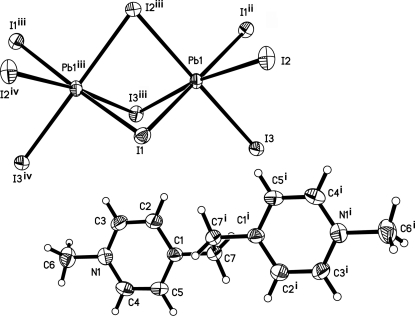
The structure of the title compound. Displacement ellipsoids are drawn at the 30% probability level. [Symmetry codes: (i) 1-x, -y, 1-z; (ii) x, 1/2-y, -1/2+z; (iii) x, 1/2-y, 1/2+z; (iv) x, y, 1+z.]

## supplementary crystallographic information

### Comment

Organic–inorganic hybrid materials offer an important opportunity to combine useful properties of inorganic and organic systems within a single molecular-scale composite (Manjunatha *et al.*, 2011). As a member of this family, there continues to be interest in the study on the design and synthesis of novel metal halides due to their potential applications in the fields of optics and electrical conductivity, as well as their structural diversity (Jin *et al.*, 2011). In this contribution, we use 1,1'-dimethyl-4,4'-(ethane-1,2-diyl)dipyridinium dichloride as an organic ligand, generating an organic-inorganic hybrid, which is reported here.

The title compound consists of discrete 1,1'-dimethyl-4,4'-(ethane-1,2-diyl)dipyridinium cations and one-dimensional ploymeric anions, [Pb_2_I_6_]_n_. The organic cation has an inversion center at the middle of the ethane C—C bond. In the anion, the Pb^II^ atom is coordinated by six iodide atoms in a distorted octahedral geometry (Fig. 1). The Pb—I bond lengths lie in a normal range (Table 1) (Lemmerer & Billing, 2006). The iodide atoms bridge the Pb^II^ atoms, forming polymeric chains running along [0 0 1]. These inorganic chains are separated by the isolated organic cations.

### Experimental

A mixture of 1,1'-dimethyl-4,4'-(ethane-1,2-diyl)dipyridinium dichloride (0.1 mmol, 0.030 g), KI (0.6 mmol, 0.10 g) and Pb(NO_3_)_2_ (0.2 mmol, 0.0662 g) in distilled water (12 ml) was placed in a Teflon-lined stainless steel vessel, heated to 423 K for 4 d and then cooled to room temperature over 12 h. Black block crystals were obtained after five months by slow evaporation of the solvent (yield: 62% based on Pb).

### Refinement

H atoms were positioned geometrically and refined as riding atoms, with C—H = 0.93 (CH), 0.97 (CH_2_) and 0.96 (CH_3_) Å and *U*_iso_(H) = 1.2(1.5 for methyl)*U*_eq_(C).

### Crystal data

**Table:**

(C_14_H_18_N_2_)[Pb_2_I_6_]	*F*(000) = 1196
*M**_r_* = 1390.08	*D*_x_ = 3.298 Mg m^−^^3^
Monoclinic, *P*2_1_/*c*	Mo *K*α radiation, λ = 0.71073 Å
Hall symbol: -P 2ybc	Cell parameters from 3425 reflections
*a* = 10.120 (6) Å	θ = 1.0–28.2°
*b* = 17.575 (10) Å	µ = 18.63 mm^−^^1^
*c* = 8.025 (4) Å	*T* = 296 K
β = 101.239 (10)°	Block, black
*V* = 1399.9 (13) Å^3^	0.25 × 0.20 × 0.19 mm
*Z* = 2	

### Data collection

**Table:**

Bruker APEXII CCD diffractometer	3425 independent reflections
Radiation source: fine-focus sealed tube	2838 reflections with *I* > 2σ(*I*)
graphite	*R*_int_ = 0.038
φ and ω scans	θ_max_ = 28.2°, θ_min_ = 2.6°
Absorption correction: multi-scan (*SADABS*; Bruker, 2001)	*h* = −13→13
*T*_min_ = 0.016, *T*_max_ = 0.030	*k* = −23→23
22768 measured reflections	*l* = −10→10

### Refinement

**Table:**

Refinement on *F*^2^	Primary atom site location: structure-invariant direct methods
Least-squares matrix: full	Secondary atom site location: difference Fourier map
*R*[*F*^2^ > 2σ(*F*^2^)] = 0.022	Hydrogen site location: inferred from neighbouring sites
*wR*(*F*^2^) = 0.046	H-atom parameters constrained
*S* = 1.02	*w* = 1/[σ^2^(*F*_o_^2^) + (0.0121*P*)^2^ + 2.6844*P*] where *P* = (*F*_o_^2^ + 2*F*_c_^2^)/3
3425 reflections	(Δ/σ)_max_ = 0.002
109 parameters	Δρ_max_ = 0.79 e Å^−^^3^
0 restraints	Δρ_min_ = −1.16 e Å^−^^3^

### Fractional atomic coordinates and isotropic or equivalent isotropic displacement parameters (Å^2^)

**Table:**

	*x*	*y*	*z*	*U*_iso_*/*U*_eq_	
C1	0.3721 (5)	0.0444 (3)	0.6192 (6)	0.0475 (11)	
C2	0.4292 (6)	0.1058 (4)	0.7117 (8)	0.0714 (17)	
H2	0.5030	0.1300	0.6817	0.086*	
C3	0.3793 (6)	0.1318 (4)	0.8467 (8)	0.0678 (16)	
H3	0.4190	0.1734	0.9084	0.081*	
C4	0.2127 (5)	0.0402 (3)	0.7986 (7)	0.0542 (13)	
H4	0.1365	0.0184	0.8274	0.065*	
C5	0.2599 (5)	0.0132 (3)	0.6641 (7)	0.0495 (12)	
H5	0.2158	−0.0269	0.6011	0.059*	
C6	0.2247 (6)	0.1251 (4)	1.0451 (8)	0.0715 (17)	
H6A	0.1488	0.0950	1.0604	0.107*	
H6B	0.1983	0.1775	1.0303	0.107*	
H6C	0.2955	0.1202	1.1433	0.107*	
C7	0.4287 (5)	0.0148 (3)	0.4740 (6)	0.0538 (12)	
H7A	0.4275	0.0552	0.3913	0.065*	
H7B	0.3715	−0.0259	0.4194	0.065*	
N1	0.2741 (4)	0.0979 (2)	0.8906 (5)	0.0521 (10)	
I1	0.84206 (3)	0.106359 (17)	0.85470 (4)	0.04934 (8)	
I2	1.04264 (4)	0.16980 (2)	0.40344 (5)	0.06218 (11)	
I3	0.60383 (3)	0.190241 (18)	0.29726 (4)	0.04830 (8)	
Pb1	0.835213 (18)	0.251424 (10)	0.60612 (2)	0.04107 (5)	

### Atomic displacement parameters (Å^2^)

**Table:**

	*U*^11^	*U*^22^	*U*^33^	*U*^12^	*U*^13^	*U*^23^
C1	0.043 (3)	0.050 (3)	0.047 (3)	−0.010 (2)	0.003 (2)	0.003 (2)
C2	0.060 (3)	0.092 (4)	0.068 (4)	−0.031 (3)	0.027 (3)	−0.018 (3)
C3	0.059 (3)	0.084 (4)	0.064 (4)	−0.027 (3)	0.019 (3)	−0.027 (3)
C4	0.041 (3)	0.052 (3)	0.072 (4)	−0.003 (2)	0.016 (3)	0.009 (3)
C5	0.043 (3)	0.040 (2)	0.067 (3)	−0.0040 (19)	0.012 (2)	0.000 (2)
C6	0.054 (3)	0.102 (5)	0.061 (4)	0.011 (3)	0.019 (3)	−0.008 (3)
C7	0.053 (3)	0.064 (3)	0.044 (3)	−0.017 (2)	0.008 (2)	−0.004 (2)
N1	0.045 (2)	0.063 (3)	0.048 (3)	0.0076 (19)	0.0089 (19)	0.003 (2)
I1	0.05674 (19)	0.04184 (15)	0.04717 (19)	−0.00310 (13)	0.00453 (14)	−0.00125 (13)
I2	0.0597 (2)	0.0799 (2)	0.0471 (2)	0.02966 (18)	0.01069 (16)	0.00598 (17)
I3	0.04488 (17)	0.05507 (18)	0.04462 (18)	−0.00848 (13)	0.00792 (13)	0.00162 (14)
Pb1	0.04505 (10)	0.04862 (10)	0.02984 (9)	−0.00104 (7)	0.00803 (7)	−0.00048 (7)

### Geometric parameters (Å, °)

**Table:**

C1—C5	1.371 (6)	C6—H6A	0.9600
C1—C2	1.372 (7)	C6—H6B	0.9600
C1—C7	1.489 (7)	C6—H6C	0.9600
C2—C3	1.361 (8)	C7—C7^i^	1.514 (10)
C2—H2	0.9300	C7—H7A	0.9700
C3—N1	1.326 (7)	C7—H7B	0.9700
C3—H3	0.9300	Pb1—I1	3.2296 (13)
C4—N1	1.334 (6)	Pb1—I1^ii^	3.2214 (13)
C4—C5	1.349 (7)	Pb1—I2	3.2311 (11)
C4—H4	0.9300	Pb1—I2^iii^	3.1724 (11)
C5—H5	0.9300	Pb1—I3	3.2435 (12)
C6—N1	1.502 (7)	Pb1—I3^iii^	3.2073 (12)
			
C5—C1—C2	117.1 (5)	C1—C7—H7B	108.9
C5—C1—C7	122.1 (4)	C7^i^—C7—H7B	108.9
C2—C1—C7	120.8 (4)	H7A—C7—H7B	107.7
C3—C2—C1	120.8 (5)	C3—N1—C4	120.4 (5)
C3—C2—H2	119.6	C3—N1—C6	119.3 (5)
C1—C2—H2	119.6	C4—N1—C6	120.3 (4)
N1—C3—C2	120.2 (5)	Pb1^iii^—I1—Pb1	76.93 (4)
N1—C3—H3	119.9	Pb1^ii^—I2—Pb1	77.60 (4)
C2—C3—H3	119.9	Pb1^ii^—I3—Pb1	76.93 (4)
N1—C4—C5	120.7 (5)	I2^iii^—Pb1—I3^iii^	86.47 (4)
N1—C4—H4	119.6	I2^iii^—Pb1—I1^ii^	92.33 (4)
C5—C4—H4	119.6	I3^iii^—Pb1—I1^ii^	99.11 (3)
C4—C5—C1	120.7 (5)	I2^iii^—Pb1—I1	87.05 (4)
C4—C5—H5	119.6	I3^iii^—Pb1—I1	83.49 (3)
C1—C5—H5	119.6	I1^ii^—Pb1—I1	177.283 (13)
N1—C6—H6A	109.5	I2^iii^—Pb1—I2	99.95 (4)
N1—C6—H6B	109.5	I3^iii^—Pb1—I2	171.539 (12)
H6A—C6—H6B	109.5	I1^ii^—Pb1—I2	86.20 (3)
N1—C6—H6C	109.5	I1—Pb1—I2	91.30 (1)
H6A—C6—H6C	109.5	I2^iii^—Pb1—I3	173.097 (12)
H6B—C6—H6C	109.5	I3^iii^—Pb1—I3	89.19 (4)
C1—C7—C7^i^	113.3 (5)	I1^ii^—Pb1—I3	83.05 (3)
C1—C7—H7A	108.9	I1—Pb1—I3	97.80 (1)
C7^i^—C7—H7A	108.9	I2—Pb1—I3	84.91 (1)
			
C5—C1—C2—C3	3.0 (9)	Pb1^iii^—I1—Pb1—I2^iii^	41.42 (2)
C7—C1—C2—C3	−178.5 (6)	Pb1^iii^—I1—Pb1—I3^iii^	−45.363 (18)
C1—C2—C3—N1	−0.1 (10)	Pb1^iii^—I1—Pb1—I2	141.321 (18)
N1—C4—C5—C1	0.1 (8)	Pb1^iii^—I1—Pb1—I3	−133.64 (2)
C2—C1—C5—C4	−3.0 (8)	Pb1^ii^—I2—Pb1—I2^iii^	−133.01 (3)
C7—C1—C5—C4	178.6 (5)	Pb1^ii^—I2—Pb1—I1^ii^	−41.307 (16)
C5—C1—C7—C7^i^	−118.8 (6)	Pb1^ii^—I2—Pb1—I1	139.759 (16)
C2—C1—C7—C7^i^	62.9 (8)	Pb1^ii^—I2—Pb1—I3	42.04 (2)
C2—C3—N1—C4	−3.0 (9)	Pb1^ii^—I3—Pb1—I3^iii^	144.45 (2)
C2—C3—N1—C6	177.1 (6)	Pb1^ii^—I3—Pb1—I1^ii^	45.168 (14)
C5—C4—N1—C3	3.0 (8)	Pb1^ii^—I3—Pb1—I1	−132.231 (15)
C5—C4—N1—C6	−177.1 (5)	Pb1^ii^—I3—Pb1—I2	−41.62 (2)

Symmetry codes: (i) −*x*+1, −*y*, −*z*+1; (ii) *x*, −*y*+1/2, *z*−1/2; (iii) *x*, −*y*+1/2, *z*+1/2.
